# Supplementary material for: MYC Deregulation and PTEN Loss Model Tumor and Stromal Heterogeneity of Aggressive Triple-Negative Breast Cancer
Source: Nat Commun. 2023 Sep 13;14:5665. doi: 10.1038/s41467-023-40841-6 (PMC10499828; doi:10.1038/s41467-023-40841-6)
Supplement: Supplementary file 1 — Supplementary Information [file 41467_2023_40841_MOESM1_ESM.pdf]

Supplemental Figure S1

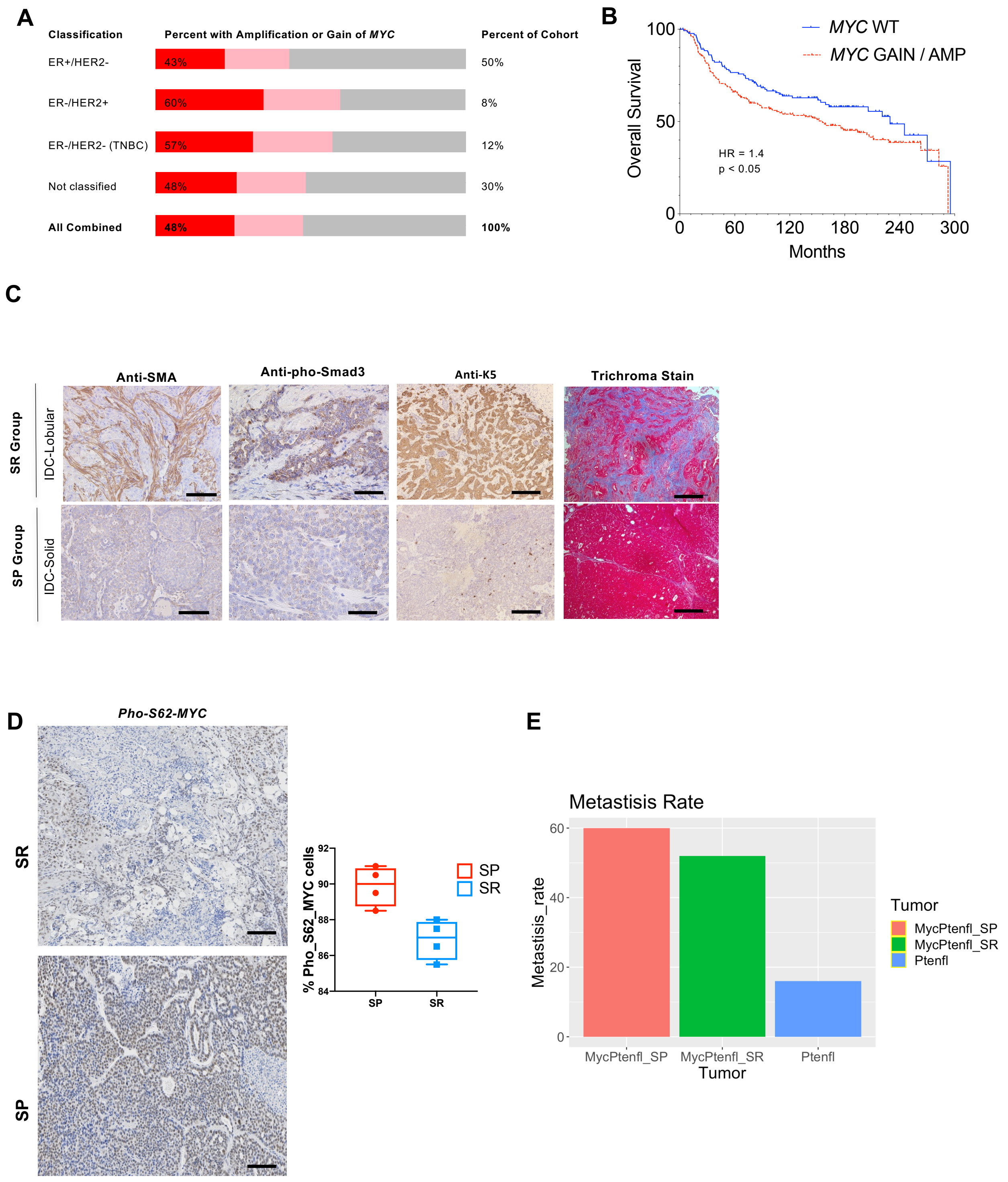

Supplemental Figure S1: MYC CNA in human breast cancer and characterization of Myc;Ptenfl subtype tumors. **A.** Low-level gain and high-level amplification of *MYC* in the METABRIC dataset [31], which includes over 2,400 primary breast cancer tumors. **B**. Overall survival between *MYC* wildtype diploid (N= 184) and MYC CNA (141) from the METABRIC dataset, (p value = 0.0350 using Gehan-Breslow-Wilcoxon test)**. C.** Representative immunohistochemical staining for the indicated proteins and Trichrome staining for collagen in stromal-rich (SR) tumors (N=20/20 positive for SMA, pSMAD3, Cytokeratin 5 (KRT5), high collagen) and stromal-poor (SP) tumors (N=10/10 negative for SMA, pSMAD3, low for KRT5 and collagen). Scale bars = 100 (SMA), 50 (pSMAD3), 100 (KRT5), 200 (Trichroma) µm. **D**. On the left; Immunohistochemistry (IHC) staining with anti-phospho-S62-MYC in stromal-rich (SR) vs stromal-poor (SP) tumors, Scale bar= 50µm. On the right; the percentage of phospho-S62-MYC positive cells were analyzed in stromal-rich tumors (N=4) compared to stromal-poor tumors (N=4), box- plots show median and interquartile range. The centerline of the boxplots represents the median value (50th percentile) and encapsulates the range from the 25th to the 75th percentiles of the dataset. The whiskers extend from the minimum to the maximum values, showcasing the full spread of the data. **E.** total metastasis rate for Ptenfl: 3/19=16%; Myc;Ptenfl SR: Macro 9/23=39%, Micro 3/23=13%; Myc;Ptenfl SP: Macro 5/15=33.3%, Micro 4/15= 26.7%.

Supplemental Figure S2

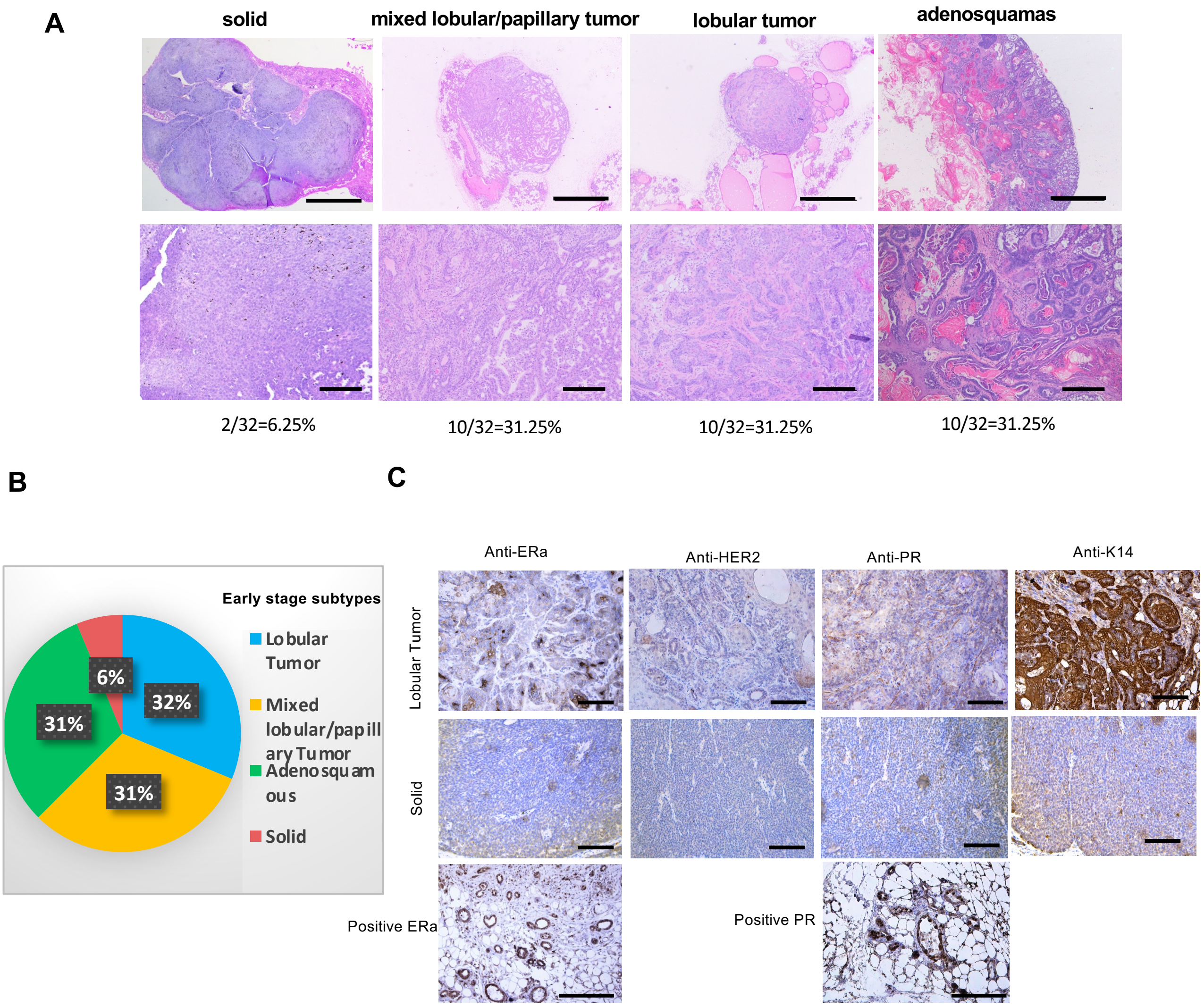

Supplemental Figure S2: Early-stage Myc;Ptenfl tumors are triple-negative and show heterogeneous histopathology. **A.** Multiple histologic subtypes present early in small tumors (dimeter <3mm) reproduced in 32 Myc;Ptenfl mice. Top panel Scale bar =1mm, bottom panel scale bar =200um. **B.** Pie chart of the frequency of histological subtypes in 32 small tumors from Myc;Ptenfl mice. **C.** On the left, Immnuhistochemistry staining of Myc;Ptenfl early-stage tumors with anti-ERa, anti-HER2, and anti-PR in papillary (N=10/10 negative), squamous (N=10/10 negative), lobular (N=10/10 negative) subtype tumors and solid tumors (N=10/10 negative); positive nuclear ERa and PR staining in adjacent normal ducts indicated below. On right, Immnuhistochemistry staining of Myc;Ptenfl early-stage tumors with the basal marker KRT14 in papillary (N=10/10 positive), squamous (N=10/10 positive), lobular (N=10/10 positive) subtype tumors and solid tumors (N=10/10 negative). Scale bars = 100 μm.

Supplemental Figure S3

A

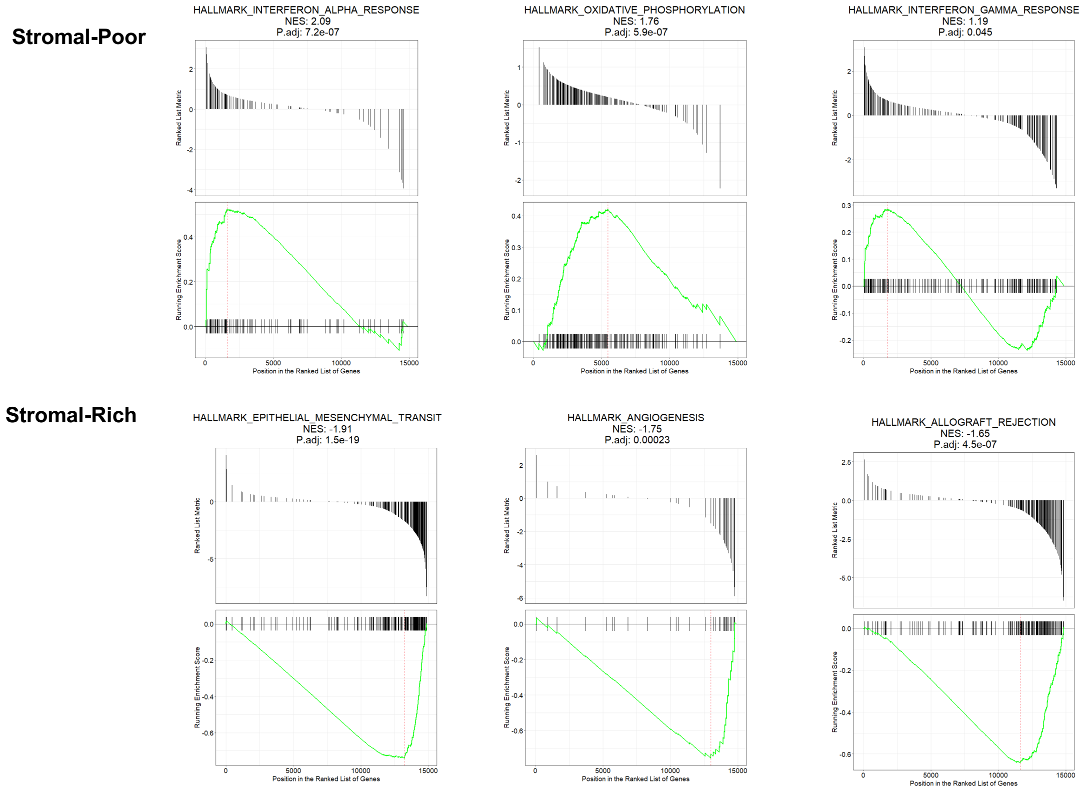

B

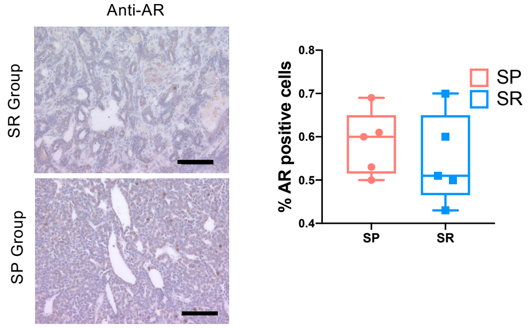

Supplemental Figure S3: Hallmark pathways active in Myc;Ptenfl subtype tumors. **A**. Gene set enrichment analysis (GSEA) comparing Myc-Ptenfl subtypes; stromal-poor (SP) tumors versus stromal-rich (SR) tumors, showing the top **3** hallmarks upregulated in each subtype. **B.** On the left; Immunohistochemistry (IHC) staining with anti-AR in stromal-rich vs stromal-poor tumors, scale bar= 100µm. On the right; the percentage of AR positive cells were quantified in stromal-rich tumors (N=5) compared to stromal-poor tumors (N=5), box-plots show median and interquartile range.

Supplemental Figure S4

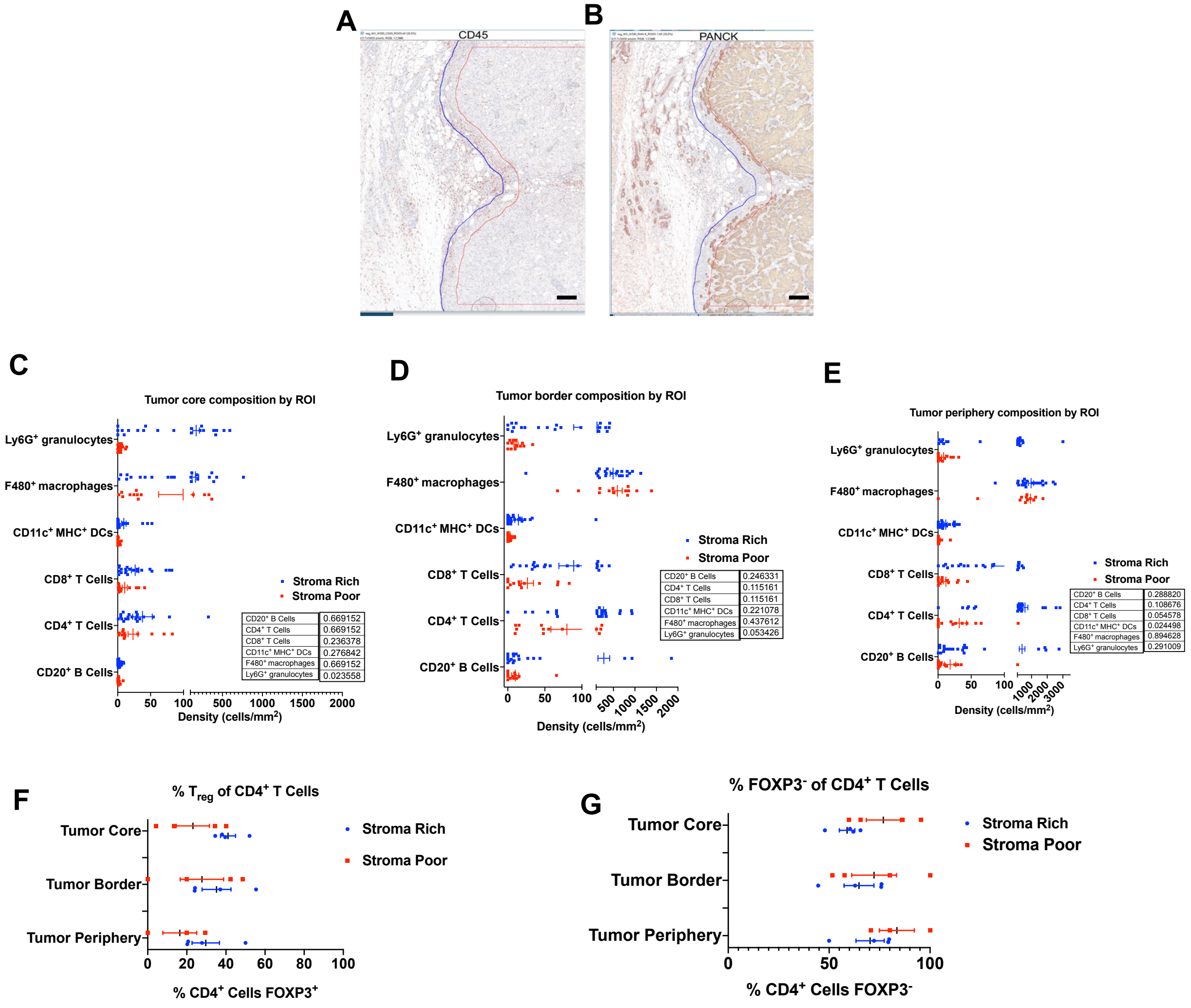

Supplemental Figure S4: Multiplex-immunohistochemistry mIHC to analyze the immune contexture of Myc;Ptenfl tumor subtypes. A. Example image of CD45 stain in Myc;Ptenfl tumor indicating tumor, border and periphery. **B.** PANCK expression was used to determine tumor boundary. **A-B.** Scale bars = 51,000 µm. **C,D,E.** The distribution of each lineage of immune population in stromal-rich compared to stromal-poor tumors; core, border, and periphery, respectively **F.** Total Treg $CD4^{+}$ cells with $Foxp3^{+}$ population in stromal-rich tumors compared to stromal-poor tumors regionally. **G.** Non-Treg $CD4^{+}$ cells in the stromal-poor tumors compared to stromal-rich regionally. **C-G.** box-and-whisker plots show median and interquartile range, p value analysis using two-way ANOVA (mixed model), the centerline of the boxplots represents the median value (50th percentile), and the box encapsulates the range from the 25th to the 75th percentiles of the dataset. Stromal-Poor (red, N=13) and Stromal-Rich (blue, N=21).

Supplemental Figure S5

A

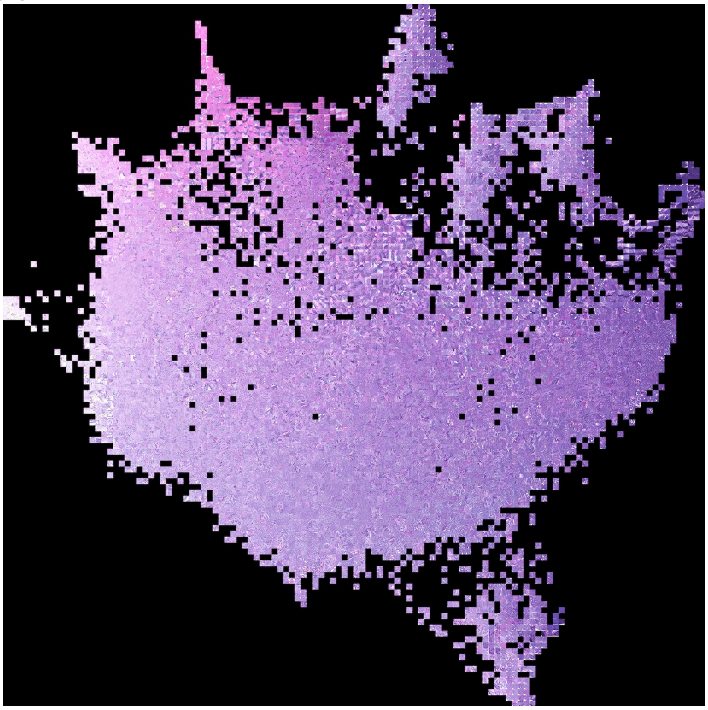

B

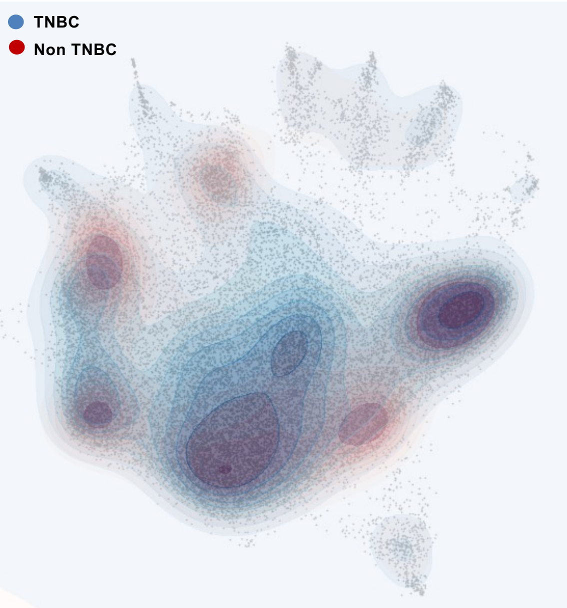

Supplemental Figure S5: Histopathological features space projection of tile images from the human breast cancer and Myc;Ptenfl mammary tumor TMAs. A. The learned VAE feature representations for each of the tiles in the TMA datasets are projected into two dimensions with the UMAP embedding. **B.** The learned VAE features based on human subtype information for each core (60% TNBC, 30% ER+ and 10 % HER2+ disease) in the human TMAs. showing both non-TNBC and TNBC subtypes overlap with each other.

Supplemental Figure S6

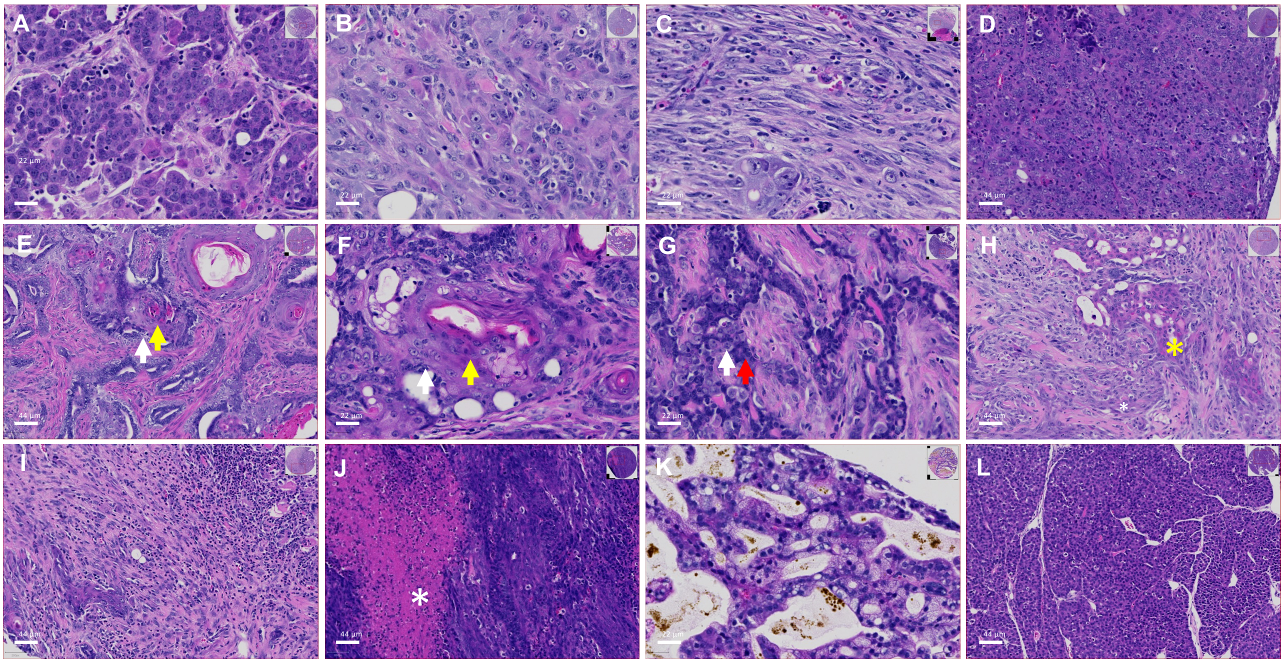

M

Histologic characteristics of human TNBC	Stromal-poor Mice		Stromal-rich Mice		(N=80)	Frequency in mice TNBC model (%)
	N= 16	Frequency (%)	N=64	Frequency (%)		
Low grade nuclear feature (A)	8	50	9	14.0625	17	21.25
High grade nuclear feature (B)	6	37.5	8	12.5	14	17.5
Sarcomatous transformation (C)	2	12.5	8	12.5	10	12.5
Solid growth pattern (D)	8	50	12	18.75	20	25
Squamoid metaplasia (E,F)	0	0	38	59.375	38	47.5
Myoepitheial proliferation (G,H)	0	0	33	51.5625	33	41.25
Geographic necrosis (J)	1	6.25	16	25	17	21.25
Fibrosis with stromal lymphocytic infiltrate (I)	2	12.5	25	39.0625	27	33.75
Clear cell change (K)	0	0	9	14.0625	9	11.25
Neuroendocrine differentiation (L)	1	6.25	0	0	1	1.25

Column scaled
High
Low

Supplemental S6: Mouse Myc;Ptenfl tumors showed many well-known human TNBC histological features. A-D. Myc;Ptenfl tumors (represent, Stromal-poor mice number = 16 and Stromal-rich mice number = 64) showed various stages of breast cancer from IDC with low-grade nuclear features (A) to high-grade nuclear features (B), sarcomatous transformation (C), and solid growth pattern (D). **E-H**. In addition, Myc;Ptenfl characteristically showed squamous-myoepithelial differentiation. The transition between squamous cells (yellow arrow) and myoepithelial cells (white arrow, E, medium magnification, F, high magnification). Luminal epithelial (red arrow) and myoepithelial (white arrow) proliferation (G). Squamous cell carcinoma (yellow asterisk) along with sarcomatous cancer (white asterisk, H). **I.** Stromal cell with inflammatory cell infiltrations. **J.** Geographic necrosis (white asterisk). **K**. Clear cell change. **L**. Neuroendocrine differentiation with thick trabeculae. Scale bars = 22 µm, 22 µm, 22 µm, 44µm, 44µm, 22µm, 22µm, 44µm, 44µm, 44µm, 22µm, 44µm, respectively. A-L. the statistics and reproducibility are shown in (Supplemental figure **S6**M). **M**. Frequency of human TNBC characteristic histologic features in Myc;Ptenfl TNBC model.

Supplemental Figure S7

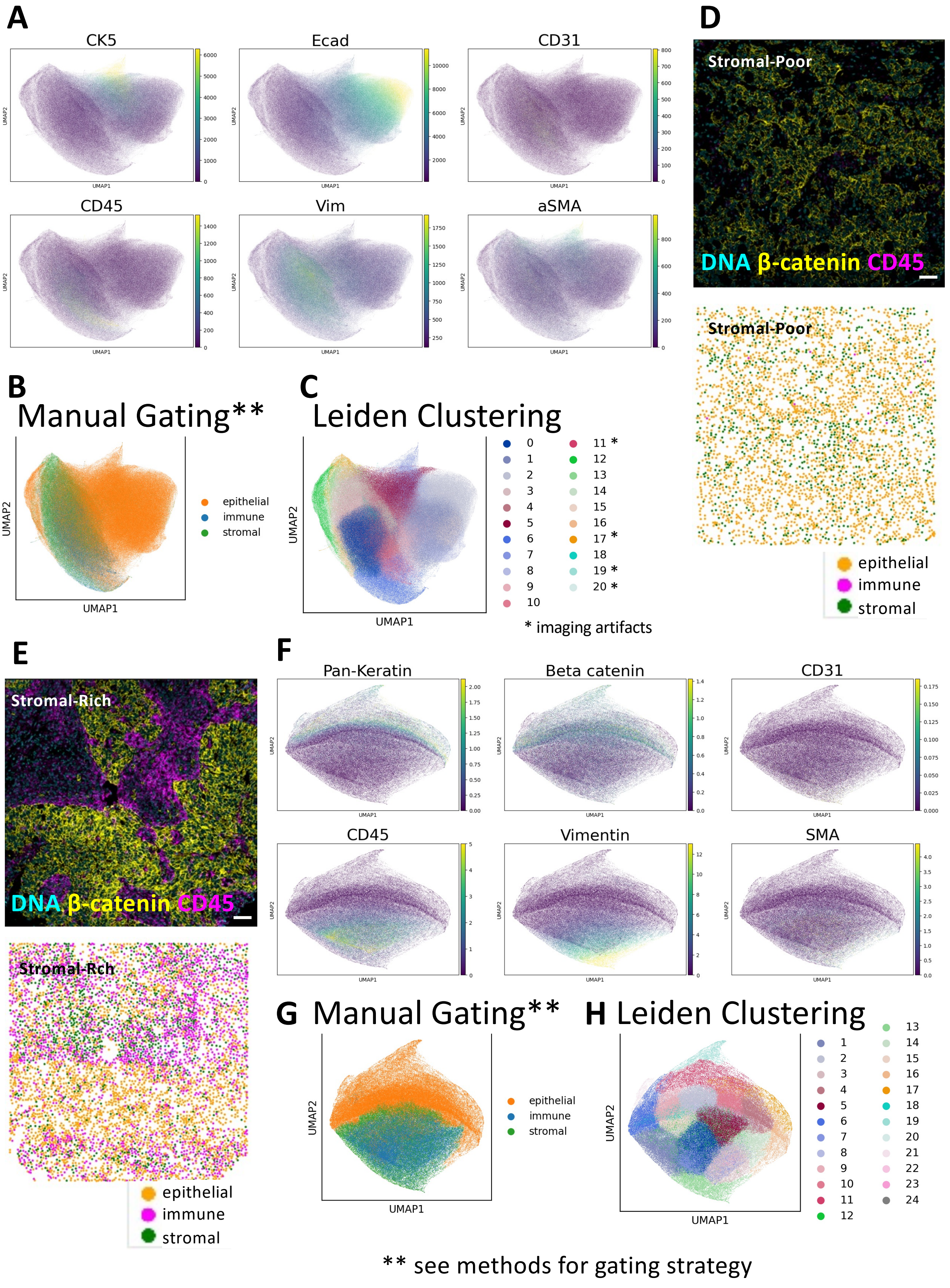

Supplemental Figure S7: Gating and unsupervised clustering-based cell type definition in Myc;Ptenfl tumor and human TNBC TMAs. A. UMAP projection of single cells in mouse TMA, colored by expression of the indicated cell type specific markers. **B.** Manual gating of mouse epithelial, immune and stromal cell types (see methods for lineage gating marker scheme) projected on the UMAP. **C.** Unsupervised clustering with the Leiden algorithm resulted in 21 cell types in mouse tumor tissues, 17 of which were subsequently annotated and 4 of which were imaging artifacts. **D-E.** Top: Representative three-color MIBI images of a 800 X 800 μm region-of-interest from stromal-poor (D) and stromal-rich subtypes (E) of human TNBC, reproducibility number are 11 and 29 respectively. Bottom: Gated cell type spatial distribution of tissues above. Orange: epithelial, pink: immune and green: stromal cells **F.** UMAP projection of single cells in human TNBC, colored by expression of cell type specific markers. **G.** Manual gating of human epithelial, immune and stromal cell types projected on the UMAP. **H.** Unsupervised clustering with the Leiden algorithm resulted in 25 cell types in human tissues.

Supplemental Figure S8

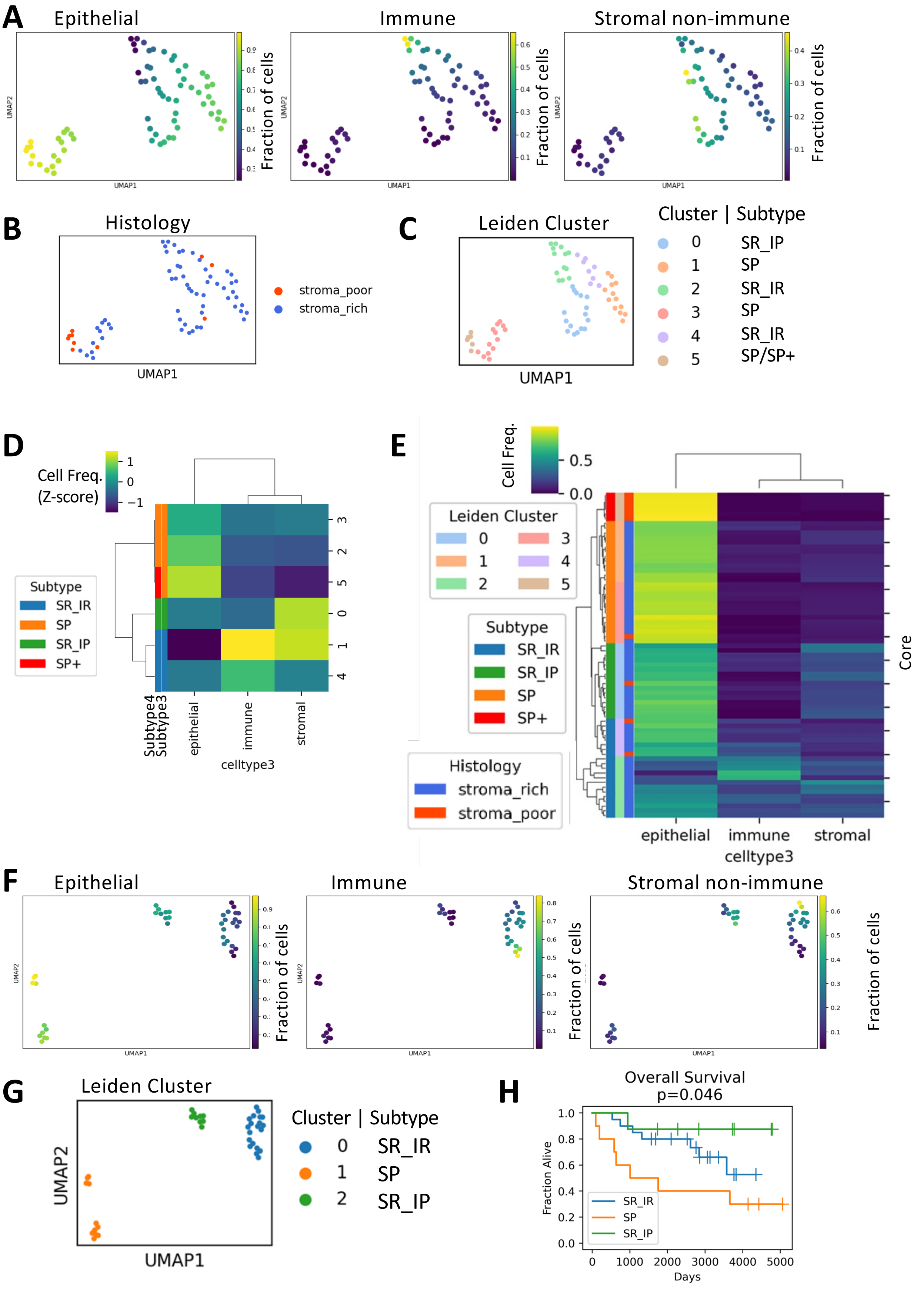

Supplemental Figure S8: Cell frequency-based subtyping of Myc;Ptenfl tumor and human TNBC tissues. A. UMAP embedding of mouse tissues based on frequencies of epithelial, immune and stromal non-immune cells in each TMA core (k-nearest neighbors k=5). **B.** Histology-based subtypes projected on same UMAP in (A). **C.** Unsupervised clustering of mouse tissues with the Leiden algorithm resulted in six clusters, annotated as three subtypes: stromal-poor (SP), stromal-rich-immune-rich (SR_IR) and stromal-rich-immune-poor (SR_IP). **D.** Hierarchical clustering of mean cell type frequency of six Leiden clusters from (C). Row annotations show meta-clusters/annotated subtypes: Very stromal-poor (SP+, red), stromal-poor (SP, orange), stromal-rich-immune-rich (SR_IR, blue) and stroma-rich-immune poor (SR_IP, green). **E.** Hierarchical clustering of mouse samples based on cell type frequency in each tumor. Row annotations show Leiden clusters, annotated subtypes, and histology subtypes. **F.** UMAP embedding of human tissues based on frequencies of epithelial, immune and stromal non-immune cells in each ROI (k-nearest neighbors k=5). **G.** Unsupervised clustering of TNBC patients using the Leiden algorithm resulted in three clusters annotates as: stromal-poor (cluster 1), stromal-rich-immune-rich (cluster 0) and stromal-rich-immune-poor subtype (cluster 2). **H**. Kaplan-Meier curves of overall survival in cell frequency-based subtypes in human TNBC. Log-rank p=0.046, n=38 patients, vertical ticks are censored patients.

Supplemental Figure S9

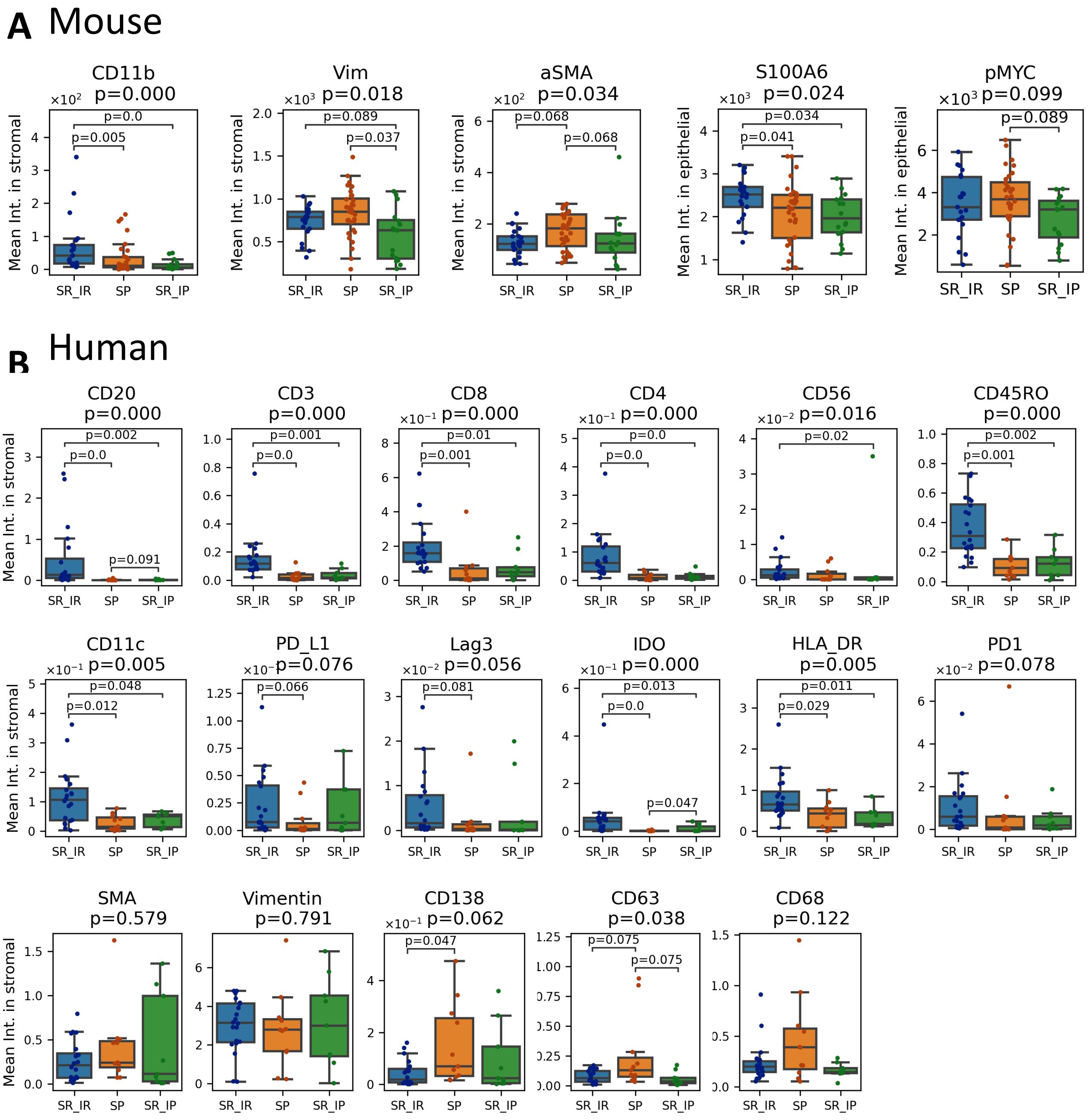

Supplemental Figure S9: Marker Expression in Cell Frequency-Based Subtypes from mouse and human TMAs. A. Stromal expression of myeloid-lineage (CD11b), activated fibroblast/pericyte (alpha-SMA), mesenchymal (Vim) markers, and epithelial mesenchymal marker S100A6 and epithelial phospho-MYC expression in mouse subtypes. **B.** Stromal expression of lymphocyte (CD20, CD3, CD8, CD4, CD56, CD138), monocyte (CD11c, CD63), immune checkpoint (PD-L1, Lag3, IDO, PD1), memory (CD45RO), antigen presentation (HLA-DR), activated fibroblast/pericyte (SMA), mesenchymal (Vimentin), and macrophage (CD68) markers in human subtypes. **A-B.** P-values determined by Kruskal–Wallis H test. Box-and-whisker plots show median and interquartile range. N=70 mouse TMA cores (A) and 40 human patients (B).

Supplemental Figure S10

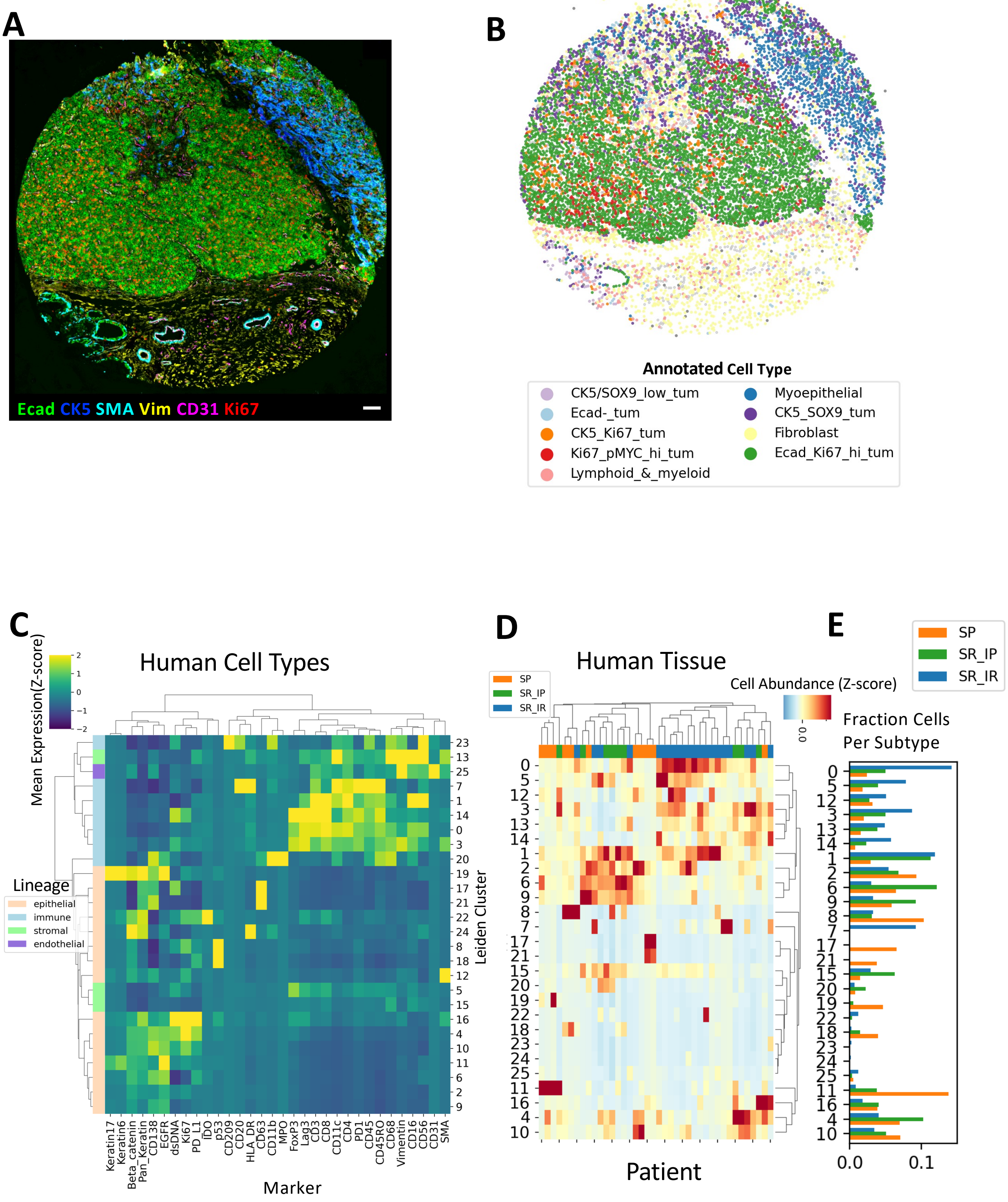

Supplemental Figure S10: Intratumoral phenotypic heterogeneity by marker expression in mouse and human TMAs. A. Six-color overlay of intratumoral and stromal heterogeneity in Myc;Ptenfl tumor cores (represent, n=59 stromal-rich mice and 10 stromal-poor mice). Scale bar is 130 µm. **B.** Unsupervised-clustering based detailed cell phenotypes reflect the spatial heterogeneity observed in (A). **C.** Mean marker intensity in each cell type defined by unsupervised Leiden clustering in human TNBC tissues. Row annotations show cell lineage: epithelial (light orange), immune (blue) or non-immune stromal (green) endothelial (purple). **D.** Hierarchical clustering of human samples based on z-scored cell phenotype abundance. Heat map column colors: stromal-poor (SP, orange), stromal-rich-immune-rich (SR_IR, blue) and stromal-rich-immune-poor (SR_IP, green). **E.** Bar plot of frequency of each cell phenotype in human subtypes.

Supplemental Figure S11

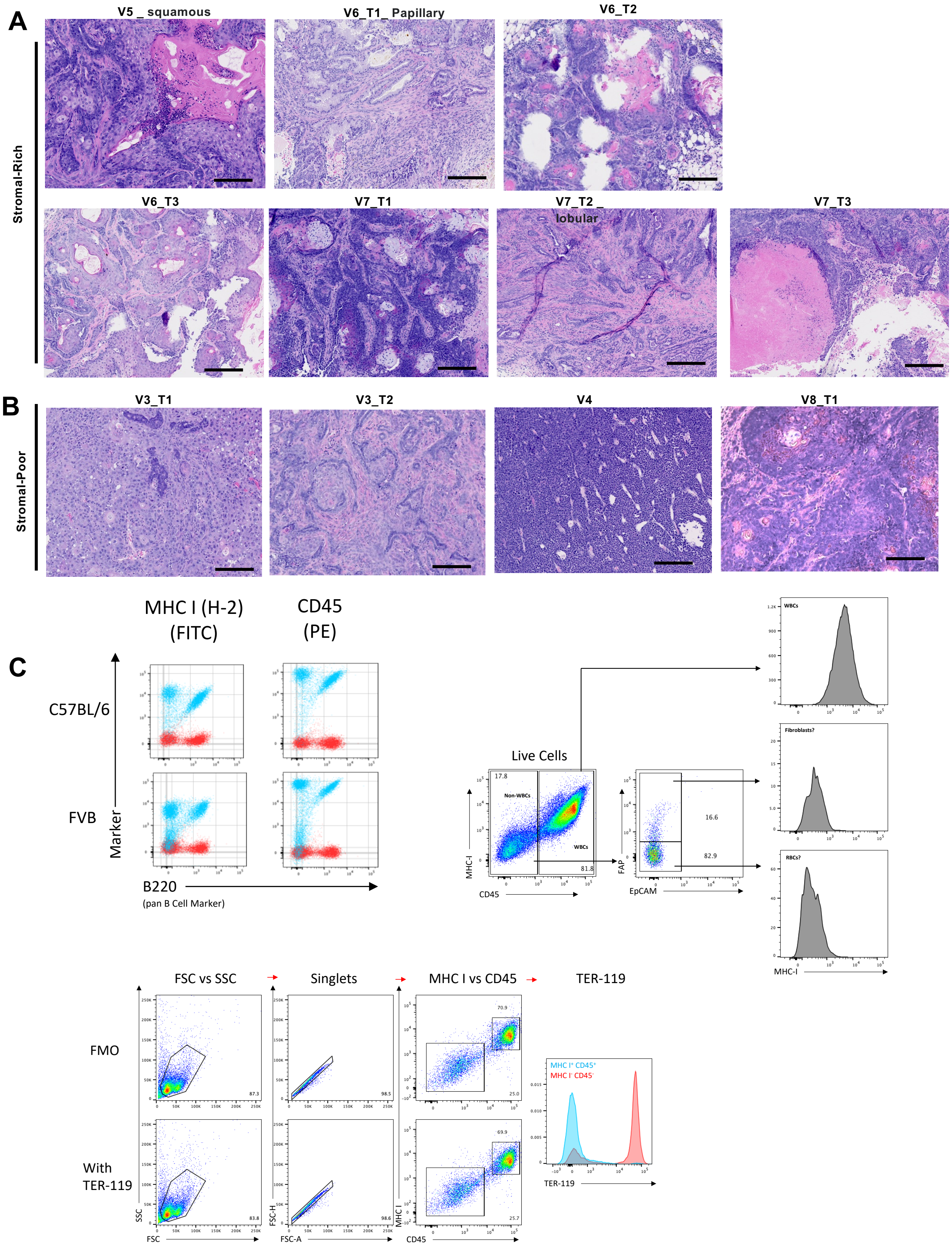

Supplemental Figure S11: Tissues and material validation used for single cell RNAseq. A-B. Histology of the 11 Myc;Ptenfl tumor samples used for scRNAseq analysis **A.** H&E showed stromal-rich histology reproduced in 7 out of 11 Myc;Ptenfl tumors. Scale bar = 100 µm. **B.** H&E showed stromal-poor solid histology reproduced in 4 out of 11 Myc;Ptenfl tumors. Scale bar = 100 µm **C.** The BioLegend TotalSeq B0301-B0306 was used, containing CD45 (clone 30-F11) and MHC-I (clone M1/42). The M1/42 clone (anti-MHC I) is reported to recognize cells from C57BL/6 (B6) mice, which have the H-2b haplotype, but it was not tested for cells from FVB mice, which have the H-2q haplotype. To test whether M1/42 binds FVB poorly, or not at all, both strains FVB and C57BL/6 mice spleen stained with fluorophore-conjugated CD45 (clone 30-F11), MHC-I (clone M1/42),EpCAM , and TER-119 (RBC marker). Thus, M1/42 cross-reacts to FVB cells, with reduced staining intensity, but was sufficiently reactive to use TotalSeq (Biolegend) reagents on FVB cells. Gating/sorting strategies: the C57BL/6 and FVB spleen stain with Markers: CD45, CD3, CD4, CD8, B220, CD11c, CD11b, Ly6C, Ly6G, TER-119, EpCAM. Then FSC/SSC adjusted so lymphocytes are centered at 50k vs. 25k. Gate on time, singlets, and cells (lymphs and larger, to edges of x and y axes) for the following: FSC/SSC of RBCs (CD3- BB20- CD11c- CD11b- TER-119+), FSC/SSC of lymphs (CD3+ vs. B220+), FSC/SSC of DCs (CD11c+ MHC II+), FSC/SSC of monos (CD11c- CD11b+ Ly6C+), FSC/SSC of PMNs (CD11c-CD11b+Ly6G+) and FSC/SSC of epithelial cells (CD45- EpCAM+).

Supplemental Figure S12

A

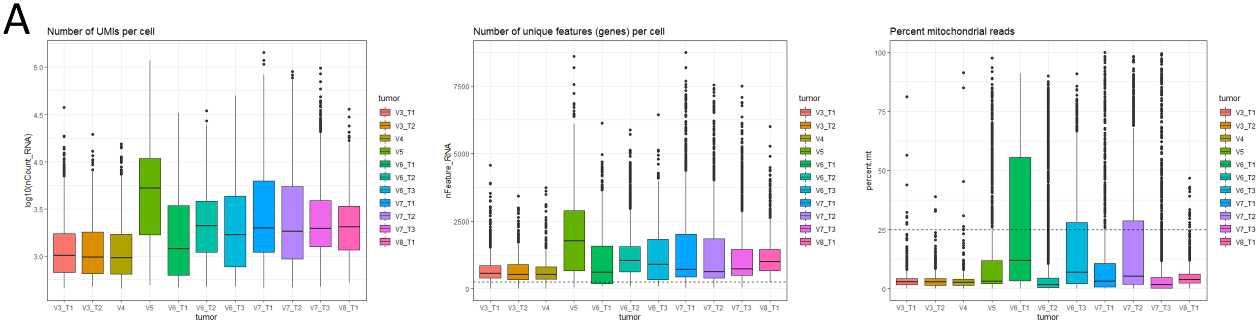

B

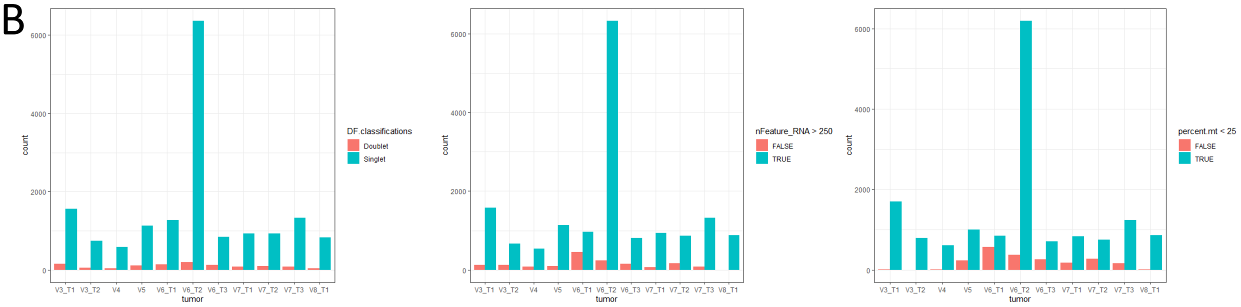

C

tumor	nCells	mean_nCount_RNA	mean_nFeature_RNA	mean_percent.mt
V3_T1	1428	1385.333	672.6092	3.504835
V3_T2	614	1467.952	691.5179	3.852925
V4	490	1466.356	680.9755	3.584096
V5	895	8792.437	2193.0559	4.367694
V6_T1	700	3117.215	1291.2300	6.954764
V6_T2	5995	2919.183	1200.7675	3.007906
V6_T3	578	3331.370	1322.6003	6.579067
V7_T1	742	5837.861	1437.7264	3.804922
V7_T2	648	5179.649	1363.6250	5.043397
V7_T3	1138	3969.579	1207.4446	2.544212
V8_T1	814	2928.386	1170.1609	4.966005

Supplemental Figure S12: scRNA-seq quality control. A. Violin plots showing QC statistics for each individual Myc;Ptenfl tumor. Boxplots line represents median value, boxes are inner quartile range (IQR) and whiskers extend to the furthest point less than 1.5*IQR from median. **B.** Number of cells passing each filtering threshold per tumor. **A-B.** Stromal-Poor tumor (N=4) and Stromal-Rich tumor (N=7). **C.** Table summarizing the number of cells, mean number of UMI recovered, mean number of unique genes, and mean percent of UMI contributed from mitochondrial mtRNA for each individual tumor.

Supplemental Figure S13

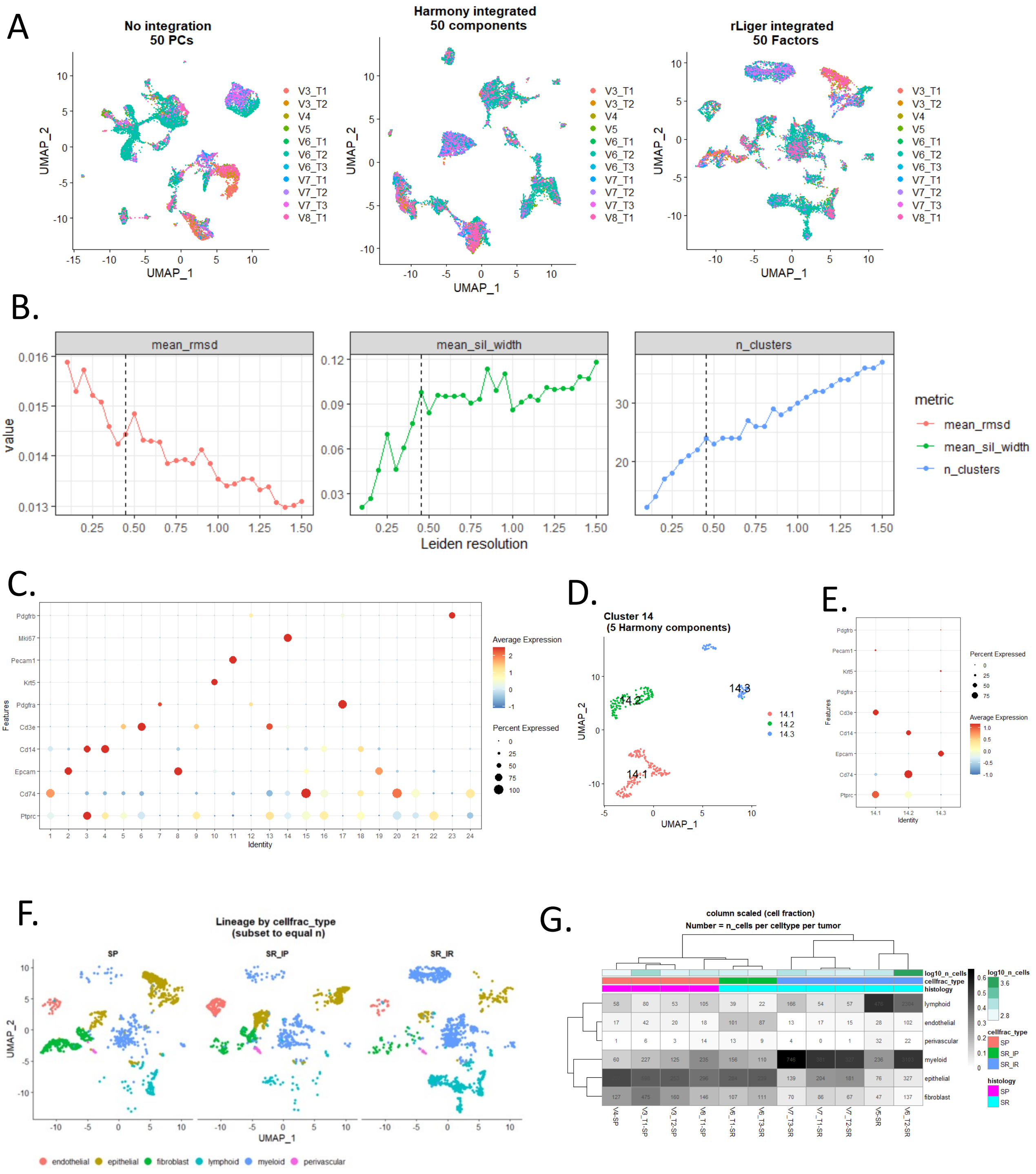

Supplemental Figure S13: iNMF data integration and clustering. A. UMAPs computed from: 50 principal components with no integration, 50 harmony integrated principal components, 50 iNMF factors computed with Rliger. Integrations were performed across scRNA-seq libraries to remove technical noise while retaining biological heterogeneity. **B.** Clustering quality results computed on the 50 iNMF factors across a range of Leiden resolutions (0.1 – 1.5). Mean RMSD and mean Silhouette Width were computed for each resolution using the R package Bluster. A resolution of 0.45 was selected as optimal and used for downstream analysis as it represented a plateau in silhouette width. **C.** Dotplot showing the average expression of select lineage related markers for each unsupervised cluster. **D.** UMAP of Cluster 14 (proliferative cluster) computed on 5 harmony components. Harmony was used to integrate the subset of cluster 14 across sequencing libraries, and 5 harmony components were chosen based on elbow plot for UMAP visualization and unsupervised clustering. **E.** Dotplot of the same lineage markers as C for cluster 14 subclusters shows that cluster 14 includes proliferating cells from three lineages (lymphoid, myeloid, and epithelial). **F.** UMAP colored by lineage and split by cellfrac_type to highlight differences in frequencies in Stromal Poor (SP), Stromal-Rich-Immune-Poor (SR-IP) and Stromal-Rich-Immune-Rich (SR-IR) tumors. UMAP was computed on 50iNMF factors. **G.** Distribution of cells found in each tumor. Heatmap color represents the proportion (fraction of total cells recovered) and inset number is the number of cells. Histology represents the two classes assigned by a pathologist (SP: Stromal Poor, SR: Stromal Rich). For Cell fraction type the stromal rich subtype was further divided into SR-IR: Stromal Rich Immune Rich, and SR-IP: Stromal Rich Immune Poor).

Supplemental Figure S14

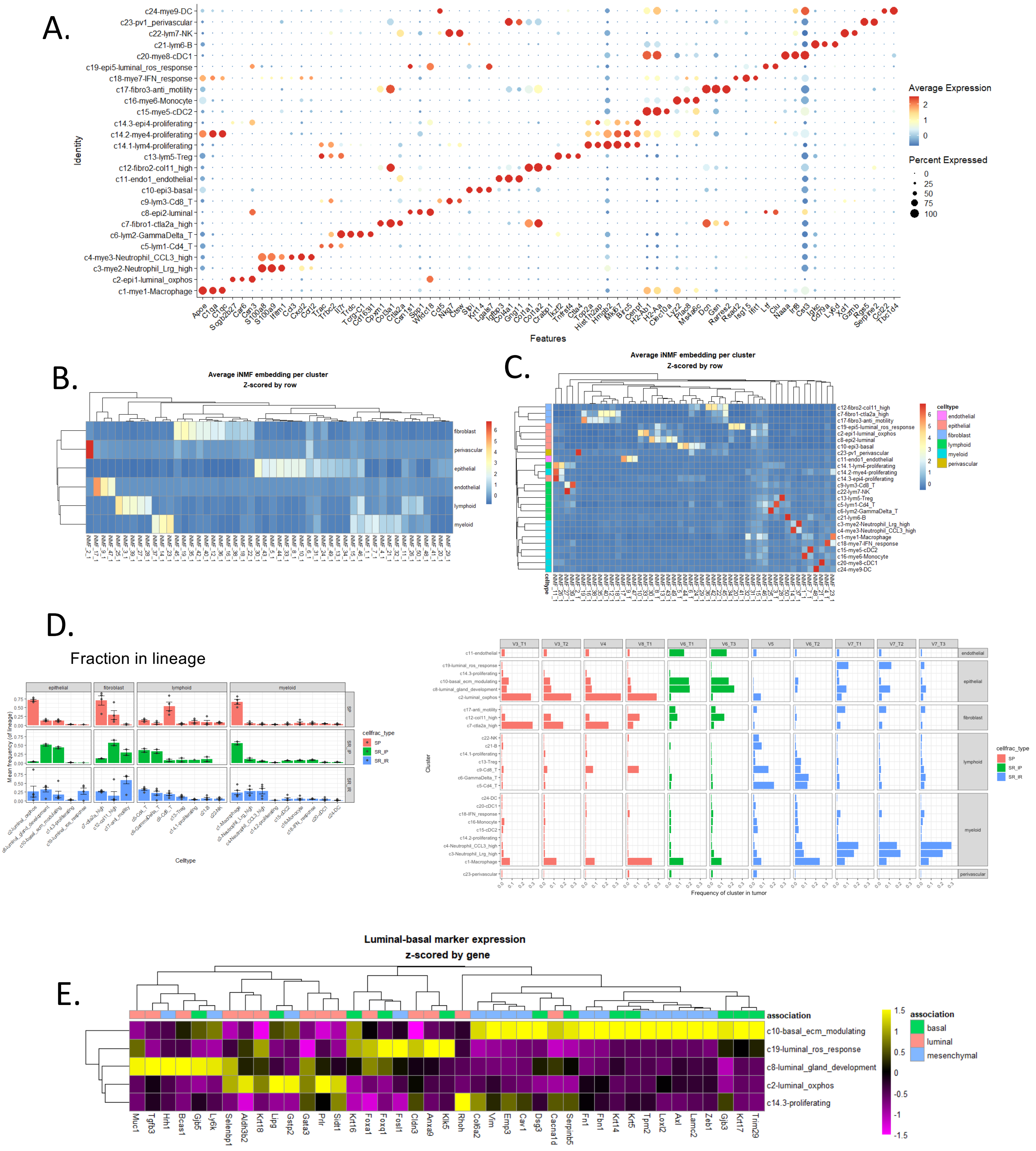

Supplemental Figure S14: iNMF data integration and clustering. A. Dotplot showing up to the top 3 differentially expressed genes for each unsupervised cluster when compared to all other clusters. **B.** Heatmap of the mean iNMF cell embedding for each lineage. Hierarchical clustering was performed on both rows and columns and is represented by dendrograms. **C.** Heatmap of the mean iNMF cell embedding for each unsupervised cluster. Hierarchical clustering was performed on both rows and columns and is represented by dendrograms. **D.** On the left: Barplot showing mean celltype frequency within lineage for each Myc;Ptenfl tumor subtype. Error bar indicates SEM. On the right: Relative proportion of each unsupervised cluster for each tumor, color coded by Myc;Ptenfl subtype. **E.** Mean cluster expression of Epithelial biomarker genes associated with basal, luminal, or mesenchymal cell states. Expression scaled to represent z scores for epithelial cells.

Supplemental Figure S15

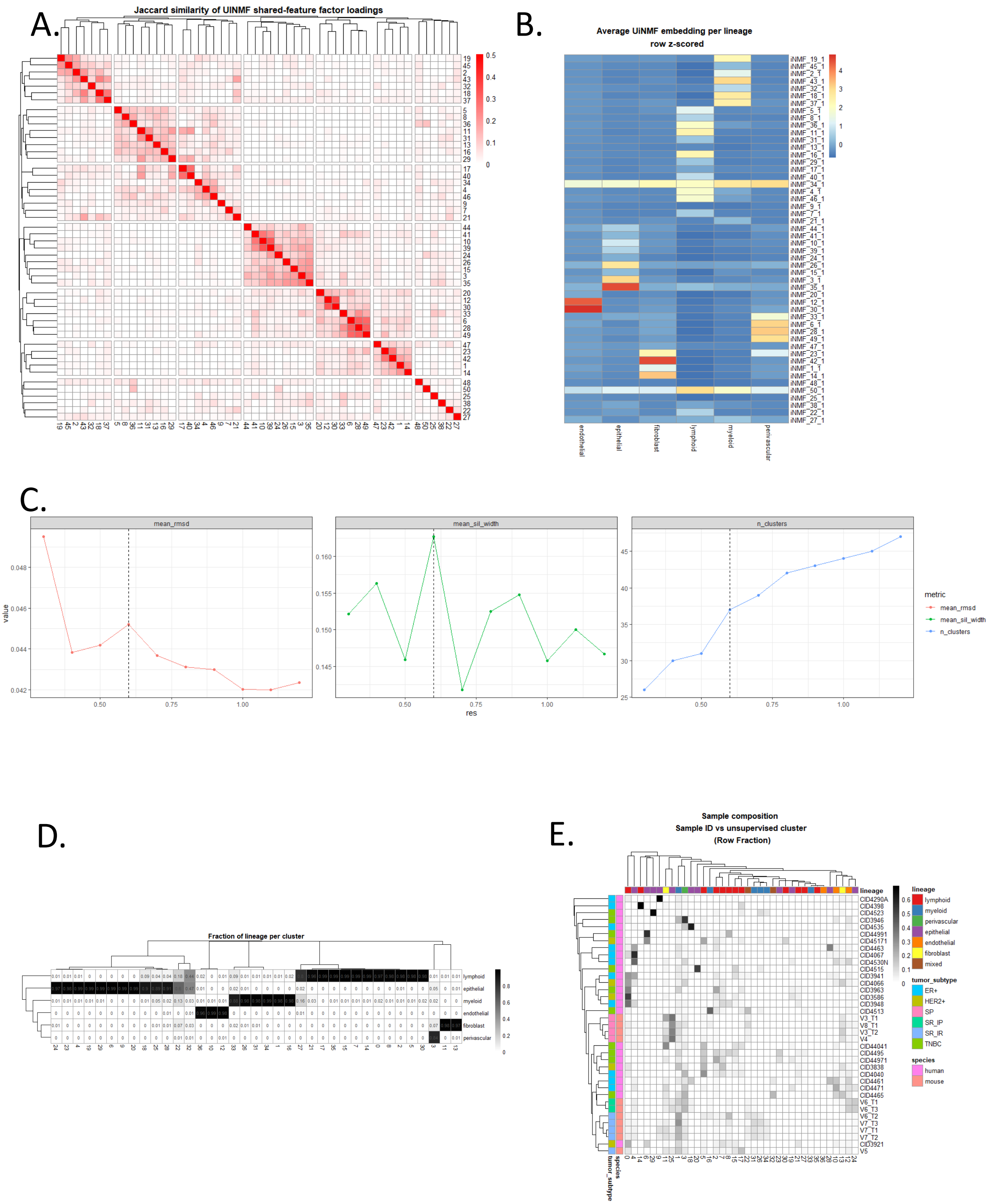

Supplemental Figure S15: Cross-species scRNA-seq comparisons. A. Jaccard similarity index computed for the top 50 weighted shared features for each UINMF factor. **B.** Mean UINMF cell embedding for each lineage computed for each factor. UINMF rows are ordered the same as figure A. **C.** Clustering quality results computed on the 50 UINMF cell embeddings across a range of Louvain resolutions (0.3 – 1.2). Mean RMSD and mean Silhouette Width were computed for each resolution using the R package Bluster. A resolution of 0.6 was selected as optimal and used for downstream analysis as it was the maximum mean silhouette width. **D.** Fraction of each lineage (assigned during single-species analysis) for each cross-species UINMF integrated cluster. **E.** Distribution unsupervised clusters present in each sample. Rows represent a unique sample (either patient from Wu et al data, or individual tumor from Myc;Ptenfl model) and columns are unsupervised clusters computed on the UINMF integrated data. Rows are annotated by species and tumor subtype (ER+, HER2+, TNBC for human data or SR-IR, SR-IP, or SP for Myc;Ptenfl data). Columns are annotated by consensus lineage as assigned in figure D. Clusters with >80% consensus were assigned their respective majority lineage, and clusters without consensus were labeled as ‘mixed’.

Supplemental Figure S16

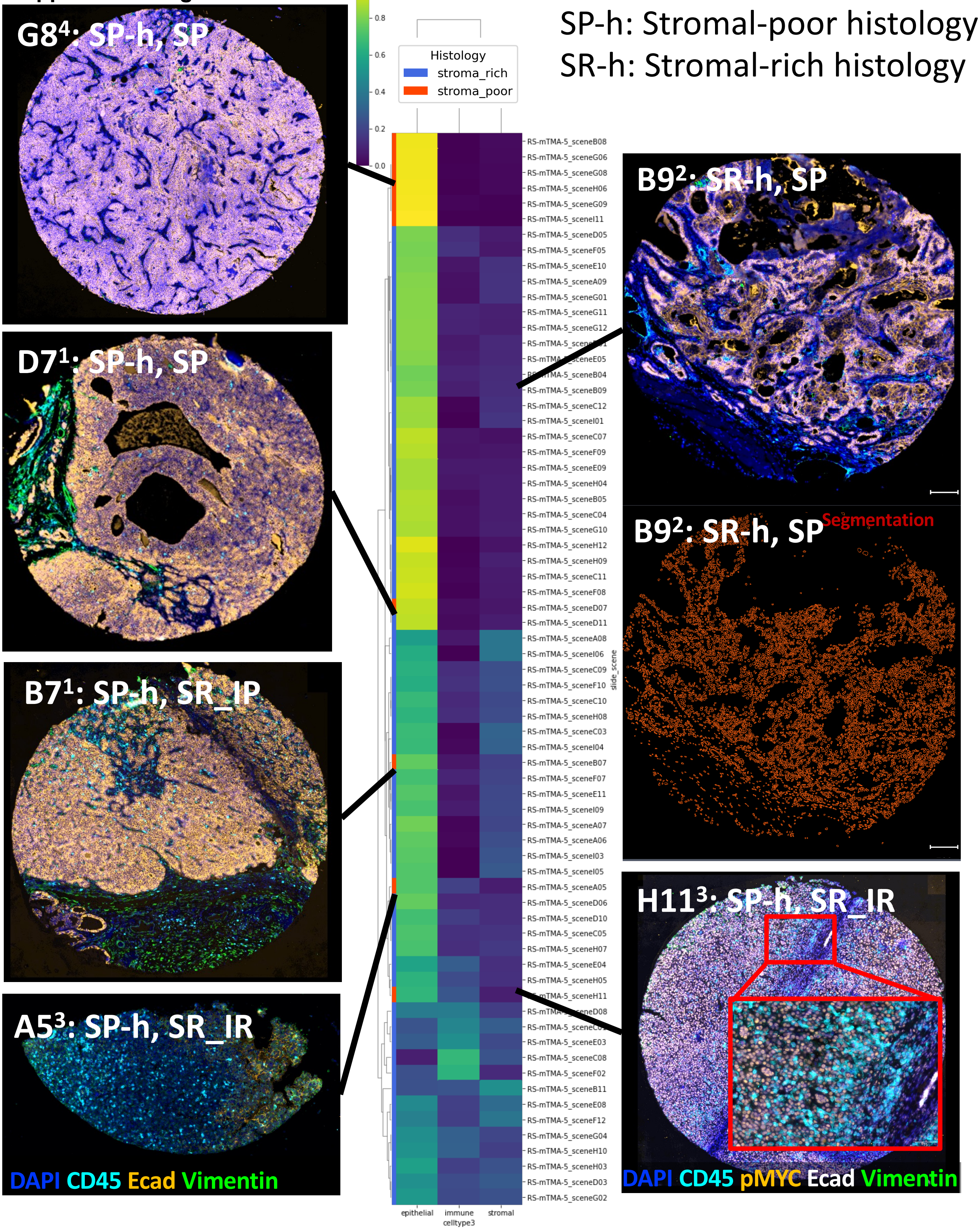

Supplemental Figure S16: Examples of discrepancies between CyCIF and histology subtypes. Central heatmap of frequency of epithelial, immune and non-immune stromal cells in CyCIF data (represent, n=59 stromal-rich mice and 10 stromal-poor mice), with rows annotated by histology subtypes. Black lines indicate examples of agreement or disagreement between CyCIF and histology, including: 1. The edge of the core includes some tumor adjacent stroma, so the SP histology gets called as SR by CyCIF (cores D7, B7). 2. The core has acellular areas with no or few cells segmented, so SR histology gets called as SP by CyCIF (Core B9). 3. There are a number of intratumoral lymphocytes that are hard to distinguish in H&E (without CD45), so SP histology becomes SR- immune-rich with CyCIF characterization (cores A5, H11). 4. Most stromal-poor by histology are also stromal-poor by CyCIF (core G8). Scale bar in core B9 is 130 µm.
