# Supplementary material for: MYC Deregulation and PTEN Loss Model Tumor and Stromal Heterogeneity of Aggressive Triple-Negative Breast Cancer
Source: Nat Commun. 2023 Sep 13;14:5665. doi: 10.1038/s41467-023-40841-6 (PMC10499828; doi:10.1038/s41467-023-40841-6)
Supplement: Supplementary file 3 — Reporting Summary [file 41467_2023_40841_MOESM3_ESM.pdf]

nature portfolio

Corresponding author(s): Rosalie Sears

Last updated by author(s): Jun 27, 2023

Reporting Summary

Nature Portfolio wishes to improve the reproducibility of the work that we publish. This form provides structure for consistency and transparency in reporting. For further information on Nature Portfolio policies, see our Editorial Policies and the Editorial Policy Checklist.

Statistics

For all statistical analyses, confirm that the following items are present in the figure legend, table legend, main text, or Methods section.

n/a	Confirmed	
☐	☒	The exact sample size (n) for each experimental group/condition, given as a discrete number and unit of measurement
☐	☒	A statement on whether measurements were taken from distinct samples or whether the same sample was measured repeatedly
☐	☒	The statistical test(s) used AND whether they are one- or two-sided<br>*Only common tests should be described solely by name; describe more complex techniques in the Methods section.*
☐	☒	A description of all covariates tested
☐	☒	A description of any assumptions or corrections, such as tests of normality and adjustment for multiple comparisons
☐	☒	A full description of the statistical parameters including central tendency (e.g. means) or other basic estimates (e.g. regression coefficient) AND variation (e.g. standard deviation) or associated estimates of uncertainty (e.g. confidence intervals)
☐	☒	For null hypothesis testing, the test statistic (e.g. F, t, r) with confidence intervals, effect sizes, degrees of freedom and P value noted<br>*Give P values as exact values whenever suitable.*
☒	☐	For Bayesian analysis, information on the choice of priors and Markov chain Monte Carlo settings
☐	☒	For hierarchical and complex designs, identification of the appropriate level for tests and full reporting of outcomes
☐	☒	Estimates of effect sizes (e.g. Cohen's d, Pearson's r), indicating how they were calculated

Our web collection on statistics for biologists contains articles on many of the points above.

Software and code

Policy information about availability of computer code

Data collection

The R package SoupX (Version: 1.6.2) was used to load the UMI gene count matrix for each library and correct for ambient mRNA contamination using default parameters (Young and Behjati, 2020). The corrected UMI gene count matrix was then converted to Seurat Object format using Seurat (v4.1.0) and the paired HTO count matrix was added. HTO demultiplexing was performed using the HTODemux function of Seurat with parameters: (kfunc = 'clara', positive.quantile = 0.95) (Satija et al., 2015; Butler et al., 2018; Stuart et al., 2019; Hao et al., 2021). Doublets were identified within each library using the R package DoubletFinder (v2.0.3) with a presumed Poisson doublet rate of 0.075 and 10 principal components(McGinnis, Murrow and Gartner, 2019). Only cells with greater than 250 unique genes expressed, less than 25% mitochondrial RNA and assigned as a 'Singlet' via DoubletFinder were retained for analysis.

Data analysis

- Code used for RNA-seq analysis is available at https://github.com/zdoha/MycPtenfl-TNBC-model
Normalized count data was generated using the standard DESeq2 (v1.36.0) workflow with Variance Stabilizing Transformation [89-91]. The top 1000 genes most variable genes were used to perform Principal Component Analysis with the "princomp" function of R package stats (v4.2.0). To normalize PCA visualization, PC embedding were scaled by dividing the embedding by the proportion of variance explained by that component. Differential expression analysis was performed with DESeq2 with LFCshrinkage via apeglm [92]. Geneset enrichment analysis (GSEA) was performed using R package Clusterprofiler [93] (v4.4.4) with the MSigDB [39] hallmark genesets, accessed via the R package msigdbr. GSEA results are detailed in Supplemental data set 1. To perform TNBC subtype correlation analysis, we first converted the 77 subtype associated human genes to murine genes using R package biomaRt (v2.52.0) [94, 95] The 60 human genes which mapped to homologous murine genes were retained, and spearman correlation was used to compare the converted and z-scored 60-gene TNBC subtype centroid signatures to MycPten;fl VST normalized and z-scored counts [12].
- Graphpad prism version 9.0.1 used for t-tests, ANOVA and survival analysis of our model.
- Cyclic IF Analysis: Code used for analysis is available at https://github.com/engjen/MYC-PTENfl-mouse. Single-cell mean intensity values of autofluorescence-subtracted images were selected from either nucleus or cytoplasm masks for each marker, depending on expected

intracellular distribution. Areas of floating tissue that created bright imaging artifacts and air bubbles that created dark artifacts were manually circled using the napari image viewer and excluded. Background subtraction was performed on markers with high background: CD31, CD45, CD8, ColIV, FoxP3, CD103, CD11b and CD11c. Filtered, background subtracted data were clustered with scanpy [90]. Two morphology features (nuclear area and nuclear eccentricity) and 20 markers were used for clustering. A Umap embedding was generated using 15 neighbors, and the Leiden algorithm was used for unsupervised clustering (resolution 0.6), resulting in 20 cell types. Inspection of cluster results in images revealed that 3 of the clusters were due to imaging artifacts and excluded. The remaining clusters were evaluated on the images and annotated. Endothelial cells were separated from mixed endothelial/immune and endothelial/fibroblast clusters by manually gating based on CD31 expression.
- Cross-species scRNAseq classifier
Orthologous genes found in both the Wu et al dataset and Myc;Ptenfl scRNA-seq data were found using the 'convert_mouse_to_human_symbols' function in NicheNetR (v1.0.0). The Wu dataset was subset to only include orthologous features, and a classifier was trained using the mixture discriminant analysis (MDA) model of scPred (v1.9.2). The classifier was then applied to the Myc;Ptenfl data with a threshold of 0.55. Adjusted Rand Index was computed with the R package aricode (v1.0.0) and used to compare the original labels derived from unsupervised clustering and transferred classes from scPred classifier.
Cross-species scRNA-seq data integration and analysis
Orthologous genes found in both the Wu et al dataset and Myc;Ptenfl scRNA-seq data were found using the 'convert_mouse_to_human_symbols' function in NicheNetR (v1.0.0) and the mouse features were updated to human symbols. The UMI count matrices for mouse and human datasets were loaded with rliger (v1.0.0) and log normalized. Variable genes were found with the 'selectGenes' function of rliger using parameters:(var.thres = 0.3, unshared = TRUE, unshared.thresh = 0.3, unshared.datasets = list(1,2)), and 1231 shared features, 268 mouse features and 1445 human features were selected. Variable features were scaled without centering in rliger, and then the optimizeALS function was run to perform matrix factorization using parameters: (lambda = 5, use.unshared = TRUE, thresh = 1e-10, k = 50, nrep = 5). The rliger 'quantile_norm' function was then used to build the shared factor graph and normalize with parameter: (ref_dataset = 'human'). The liger object was then converted to Seurat format with the rliger function 'ligerToSeurat'. The cell loadings from all 50 UINMF factors were used for UMAP embedding and unsupervised clustering. Unsupervised clustering was performed using the Louvain algorithm as implemented in Seurat, and a resolution of 0.6 was selected as it optimized for maximum mean silhouette width as estimated with the 'approxSilhouette' function of the R package Bluster (v1.2.1). Unsupervised clusters were assigned lineage if there was at least 80% agreement of prior lineage annotation of the cluster's constitutive cells, otherwise they were labeled 'mixed'.

For manuscripts utilizing custom algorithms or software that are central to the research but not yet described in published literature, software must be made available to editors and reviewers. We strongly encourage code deposition in a community repository (e.g. GitHub). See the Nature Portfolio guidelines for submitting code & software for further information.

Data

Policy information about availability of data

All manuscripts must include a data availability statement. This statement should provide the following information, where applicable:

- Accession codes, unique identifiers, or web links for publicly available datasets
- A description of any restrictions on data availability
- For clinical datasets or third party data, please ensure that the statement adheres to our policy

Data availability:
The raw bulk RNA-sequencing data generated in this study have been deposited in the Gene Expression Omnibus (GEO) database under accession number GSE215071 [ncbi.nlm.nih.gov/geo/query/acc.cgi?acc=GSE215071]. The raw single-cell RNA-sequencing data generated in this study have been deposited in the Gene Expression Omnibus (GEO) database under accession number GSE215070 [ncbi.nlm.nih.gov/geo/query/acc.cgi?acc=GSE215070].
The mIHC data generated in this study are provided in the Source Data file. The raw data of the morphological features of Myc;Ptenfl tumors generated in this study are provided in the Source Data file. The data used for Cyclic Multiplexed-Immunofluorescence in this study is available at: [https://github.com/engjen/MYC-PTENfl-mouse]. The MIBI publicly available data used in this study are available through the GitHub [https://github.com/aalokpatwa/rasp-mibi] [65]. The publicly available scRNA-seq data used in this study are available through the Gene Expression Omnibeus under accession number GSE176078 [ncbi.nlm.nih.gov/geo/query/acc.cgi?acc=GSE176078] [74]. The remaining data are available within the Article, Supplementary Information or Source Data file.

Source data are provided with this paper:
Single Source Data file is available, containing the following datasets: Bulk_msigdb_GSEA, Mice Tumor Volume (mm3), mIHC Antibodies, mTMA-Markers, Myc;Ptenfl mice TMA histologic features, and Cell type_markers used in scRNA-seq analysis.

Code availability
- RNA-seq analysis code generated for this study is available at: https://github.com/zdoha/MycPtenfl-TNBC-model.

- Variational autoencoder (VAE) code for Histologic/morphologic analysis generated for this study is available at: https://github.com/schaugf/ImageVAE

- Cyclic Multiplexed-Immunofluorescence analysis code generated for this study is available at: https://github.com/engjen/MYC-PTENfl-mouse

- R code for analysis of scRNA-seq data generated for this study can be found at: https://github.com/HeiserLab/NatureComms_MycPtenAtlas

Human research participants

Policy information about studies involving human research participants and Sex and Gender in Research.

Reporting on sex and gender	The research conducted specifically on female subjects, as breast cancer predominantly affects females. with median age at the time of breast cancer diagnosis is 62.

Population characteristics	All human tissue microarray (TMA) samples (total of 172) are surgical (mastectomy or resections) collected at Vanderbilt with the same fixation protocol. 65 surgical TNBC samples, 17 had neoadjuvant therapy. Other patients were part of adjuvant treatment.
Recruitment	Participants gave informed consent to participate in the repository and were not compensated for participation. Informed consent was obtained from all repository participants.
Ethics oversight	The Vanderbilt IRB approved the Breast Tissue and Body Fluids repository study protocol (#030747) from which patient tissues were selected for inclusion in the TMAs.

Note that full information on the approval of the study protocol must also be provided in the manuscript.

Field-specific reporting

Please select the one below that is the best fit for your research. If you are not sure, read the appropriate sections before making your selection.

☒ Life sciences ☐ Behavioural & social sciences ☐ Ecological, evolutionary & environmental sciences

For a reference copy of the document with all sections, see nature.com/documents/nr-reporting-summary-flat.pdf

Life sciences study design

All studies must disclose on these points even when the disclosure is negative.

Sample size	For the mouse work, our sample size was determined to ensure an adequate representation of each subtype. We aimed to have a minimum of 3 mice for each subtype to capture the heterogeneity present in the Stromal-Poor and Stromal-Rich subtypes. Based on our data, we observed that the Stromal-Poor subtype occurs at approximately 30% frequency, while the Stromal-Rich tumors occur at approximately 70% frequency. Therefore, by including at least 11 mice, to ensure a sufficient representation of the tumor subtypes in our study.<br><br>For the human TMA analysis, the sample size consisted of 172 TMA cores/patients. This sample size was chosen to provide a comparable and meaningful comparison with the mouse TMA dataset, which consisted of 80 mice. The aim was to establish a sufficient level of comprehensiveness and statistical power in analyzing the human samples, allowing for robust comparisons and correlations between the mouse and human data.<br><br>While we did not perform a formal statistical calculation to determine the sample size, our choices were based on considerations of capturing the relevant tumor subtypes and achieving meaningful comparisons between the mouse and human datasets. The rationale behind these sample sizes lies in striking a balance between practical feasibility and scientific significance, ensuring that we have an adequate number of samples to draw meaningful conclusions.
Data exclusions	No data were excluded.
Replication	RNA and single-cell RNA sequencing: For these experiments, we conducted replicates using independent biological samples to assess the consistency and reproducibility of our transcriptomic data. We ensured that the replication samples were obtained from different individuals or cell populations, and that the experimental procedures were performed independently. This allowed us to verify the robustness and reproducibility of our RNA sequencing results.<br><br>Multiplex immunohistochemistry (mIHC): In our mIHC experiments, we applied a replication strategy by repeating the staining and imaging process on different tissue sections from the same specimens or from distinct samples. This enabled us to validate the reproducibility of the staining patterns and the consistency of the quantitative measurements across multiple replicates.<br><br>Tissue microarray (TMA) for histology and cyclic immunofluorescent analysis: To ensure reproducibility in our histological analyses, we incorporated multiple cores from different areas of the tissue samples into the TMA. By analyzing multiple replicates of the same specimens, we could verify the consistency of the staining patterns, assess the reproducibility of the observed cellular distributions, and confirm the reliability of the cyclic immunofluorescent analysis.<br><br>In all these experimental approaches, we took measures to validate that all attempts at replication were successful
Randomization	The study design randomly assigns participant mice into an experimental group or a control group. As the study is conducted, the only expected difference between the control and experimental groups is the outcome variable being studied.
Blinding	The investigators were blinded to group allocation during histological and morphological tumor data collection

Reporting for specific materials, systems and methods

We require information from authors about some types of materials, experimental systems and methods used in many studies. Here, indicate whether each material, system or method listed is relevant to your study. If you are not sure if a list item applies to your research, read the appropriate section before selecting a response.

Materials & experimental systems

n/a	Involved in the study
☐	☒ Antibodies
☒	☐ Eukaryotic cell lines
☒	☐ Palaeontology and archaeology
☐	☒ Animals and other organisms
☒	☐ Clinical data
☒	☐ Dual use research of concern

Methods

n/a	Involved in the study
☒	☐ ChIP-seq
☐	☒ Flow cytometry
☒	☐ MRI-based neuroimaging

Antibodies

Antibodies used

HER2 (Cell Signaling #2242, 1:50); ERa (Millipore #04-227, 1:50); PR (Abcam #ab131486, 1:1000); AR (Abcam #ab47563, 1:50); cytokeratin 5 (Abcam #ab52635, 1:100); cytokeratin 14 (Covance #PRB-155P, 1:1000); pSmad3 (Abcam #ab52903, 1:100); Laminin (Abcam #ab11575, 1:50); SMA (Abcam #ab5694, 1:100); pS62 Myc rat monoclonal 4B12 [88]; Ki-67 (Abcam #15580, 1:1000); CSF-1R (Santa Cruz #sc-692, 1:500); F4/80 (Serotec A3-1, 1:200); CD11C (Cell Signaling #97585,1:100); CD4 (Cell Signaling #25229, 1:100); MHCII (eBioscience #eB14-5321, 1:100); BTK (LSBio #LS-C180161, 1:100); CD45 (BD Bioscience #550539,1:50); PDL1 (Cell Signaling #13684,1:50); CD8 (eBiosceience #14-0808082, 1:100); CD3 (Thermo #RM-9107-s, 1:300); CD207 (eBioscience #14-2073-82, 1:100); CD206 (Abcam #64693, 1:1000); B220 (BD Bioscience #550286, 1:100); RORgt (Abcam #ab207082, 1:100); Foxp3 (eBioscience #14-5773-82, 1:100); GATA3 (Abcam #ab199428, 1:100); CD11b (Abcam #ab133357,1:100); TCF1/TCF7 (Cell Signaling #2203s, 1:100); TIM3 (Cell Signaling #83882, 1:200); EOMES (Abcam #ab183991,1:1000); Granzyme B (Abcam #ab4059, 1:200); Ly6G (eBioscience #551459, 1:200); PAN Keratin (Abcam #ab27988, 1:100). CK5 (abcam, EP1601Y, 1:100); S100A6 (CST, D9F9D, 1:100); CD11c (CST, D1V9Y, 1:100); CD103 (Biolegend, 2E7, 1:100); aSMA (Santa Cruz, 1A4, 1:100); EpCAM (CST, E6V8Y); CD31 (Abcam,EPR17260, 1:100); ColVI (MDBiosciences, EPR17072, 1:100); CD11b (Abcam, EPR1344, 1:100); Ki67 (CST, D3B5, 1:100); FoxP3 (Novus, NB100-39002, 1:100); Vim (CST, D21H3, 1:100); CD45 (CST, D3F8Q, 1:100); Gal3 (Biolegend, 125408, 1:100); CollV (MDBiosciences, 203003, 1:100).

Validation

Species Compatibility: We ensured that each primary antibody used in our experiments was specifically designed and validated for the target species. This involved verifying the compatibility of the antibody with the species under investigation, thereby ensuring reliable and species-specific binding.

Application-Specific Validation: Each antibody was thoroughly validated for its intended application. We followed established protocols and best practices to assess the antibody's performance and specificity in the particular experimental techniques employed in our study.

Quality Control Measures: We employed stringent quality control measures to evaluate the performance of the primary antibodies. This involved conducting appropriate controls, including positive and negative controls, to assess specificity, sensitivity, and background levels. We also ensured that the antibody consistently produced reliable and reproducible results across multiple experiments.

Animals and other research organisms

Policy information about studies involving animals; ARRIVE guidelines recommended for reporting animal research, and Sex and Gender in Research

Laboratory animals

Rosa-LSL-Myc mice were crossed with Ptenflox/flox (Akira Suzuki et al. Immunity 2001) and Blg-Cre mice (gift from Owen Sansom, Beatson Institute for Cancer Research, Glasgow, United Kingdom) to generate mice that express MYC and deleted PTEN in response to Cre-mediated recombination in the mammary gland. The PTENfl and MYC;PTENfl mice investigated in this manuscript are in a pure FVB background.

In our study, the laboratory animals used were aged 10-12 weeks and older. Specifically, we selected female mice that had passed two cycles of pregnancy/lactation to induce Blg-Cre activation at around 10-12 weeks of age. This approach allowed us to capture a specific stage of Cre expression during late pregnancy and lactation [34].

Regarding the housing conditions, the mice were maintained at the Oregon Health & Science University Animal Care facility. The facility adheres to rigorous animal welfare standards and guidelines to ensure the well-being and proper care of the animals. The housing conditions for the mice were as follows:

Light/Dark Cycle: The mice were subjected to a 12-hour light and 12-hour dark cycle. This cycle mimics the natural light variations that the mice would experience in their native environment, providing a suitable diurnal rhythm.

Ambient Temperature: The ambient temperature in the housing facility was maintained within the range of 65-75°F (~18-23°C). This temperature range helps provide a comfortable and stable environment for the mice, minimizing any undue stress or discomfort.

Humidity: The humidity levels in the housing facility were maintained between 40-60%. This humidity range is considered optimal for the well-being of the mice, ensuring a suitable level of moisture in the air without causing excessive humidity-related issues.

Wild animals

The study did not involve wild animals

Reporting on sex	Because the Blg-cre activation of Myc expression and deletion of Pten requires lactation (expression of Blg), the MycPten;fl model only develops tumors in female mice.
Field-collected samples	no field collected samples were used in the study.
Ethics oversight	All protocols for mouse experiments described in this study were approved by the Oregon Health & Science University Animal Care and Use Committee protocol # IP00001014, Portland, OR.

Note that full information on the approval of the study protocol must also be provided in the manuscript.

Flow Cytometry

Plots

Confirm that:

☒ The axis labels state the marker and fluorochrome used (e.g. CD4-FITC).

☒ The axis scales are clearly visible. Include numbers along axes only for bottom left plot of group (a 'group' is an analysis of identical markers).

☒ All plots are contour plots with outliers or pseudocolor plots.

☒ A numerical value for number of cells or percentage (with statistics) is provided.

Methodology

Sample preparation	Single-cell suspensions were stained in FACS buffer (1X PBS, with 2% FBS and 0.1% NaN3) at 4oC, for 20 minutes, in the dark. Antibodies used are as follows: CD45 (clone 30-F11) and MHC-I (clone M1/42), EpCAM (clone G8.8) and TER-119 (clone TER-119).
Instrument	Cells were run on a BD FACS Symphony A5 (BD Biosciences).
Software	Data were analyzed using FlowJo 10.6 (Tree Star, Inc., RRID:SCR_008520).
Cell population abundance	To assess the abundance of specific cell populations, we performed flow cytometry analysis on the post-sort fractions. Cells were stained with fluorochrome-conjugated antibodies targeting specific cell surface markers. The analysis was conducted using a flow cytometer equipped with appropriate detectors and software for data acquisition and analysis.<br><br>The purity of the sorted samples was determined by evaluating the percentage of cells expressing the specific markers of interest. We established gating strategies based on the fluorescence intensity and utilized appropriate isotype controls to define the positive and negative populations accurately. By setting stringent gating criteria, we aimed to minimize contamination and ensure the purity of the sorted fractions.<br><br>Quantitative analysis was performed by calculating the percentage of cells within the sorted fractions that exhibited the desired marker expression.<br><br>It is important to note that stringent quality control measures were implemented throughout the sorting process to maintain the integrity and reliability of the data. These measures included regular instrument calibration, compensation controls, and appropriate sample handling techniques to minimize any potential artifacts or experimental variability.
Gating strategy	Regarding the gating strategy used in our experiments, we employed the following approach for the C57BL/6 and FVB spleen samples stained with the specified markers: CD45, CD3, CD4, CD8, B220, CD11c, CD11b, Ly6C, Ly6G, TER-119, and EpCAM.<br><br>First, we adjusted the forward scatter (FSC) and side scatter (SSC) parameters so that lymphocytes were centered at 50k vs. 25k. Subsequently, we applied gating steps to identify specific cell populations within the samples, and define the boundaries between "positive" and "negative" staining cell populations<br><br>The gating strategy involved sequential steps to gate on time, singlets, and cells (lymphs and larger) while ensuring proper positioning along the x and y axes. The specific gates used were as follows:<br><br>FSC/SSC gating of red blood cells (RBCs): CD3- BB20- CD11c- CD11b- TER-119+ cells were gated based on their FSC/SSC characteristics.<br><br>FSC/SSC gating of lymphocytes: CD3+ cells were distinguished from B220+ cells based on their FSC/SSC profiles.<br><br>FSC/SSC gating of dendritic cells (DCs): CD11c+ MHC II+ cells were identified using their FSC/SSC characteristics.<br><br>FSC/SSC gating of monocytes (monos): CD11c- CD11b+ Ly6C+ cells were gated based on their FSC/SSC properties.<br><br>FSC/SSC gating of polymorphonuclear cells (PMNs): CD11c- CD11b+ Ly6G+ cells were selected using their FSC/SSC characteristics.<br><br>FSC/SSC gating of epithelial cells: CD45- EpCAM+ cells were identified based on their FSC/SSC properties.

By applying these gating strategies, we were able to accurately define and distinguish the different cell populations of interest within the samples.

☒ Tick this box to confirm that a figure exemplifying the gating strategy is provided in the Supplementary Information.
